# Use of the 2-Pyridinealdoxime/N,N′-Donor Ligand Combination in Cobalt(III) Chemistry: Synthesis and Characterization of Two Cationic Mononuclear Cobalt(III) Complexes

**DOI:** 10.1155/2010/159656

**Published:** 2010-07-18

**Authors:** Konstantis F. Konidaris, Catherine P. Raptopoulou, Vassilis Psycharis, Spyros P. Perlepes, Evy Manessi-Zoupa, Theocharis C. Stamatatos

**Affiliations:** ^1^Department of Chemistry, University of Patras, 26500 Patras, Greece; ^2^Institute of Materials Science, NCSR “Demokritos”, 15310 Aghia Paraskevi Attikis, Greece

## Abstract

The use of 2-pyridinealdoxime (paoH)/N,N′-donor ligand (L-L) “blend” in cobalt chemistry has afforded two cationic mononuclear cobalt(III) complexes of the general type [Co(pao)_2_(L-L)]^+^, where L-L = 1,10-phenanthroline (phen) and 2,2′-bipyridine (bpy). The CoCl_2_/paoH/L-L (1 : 2 : 1) reaction system in MeOH gives complexes [Co^III^(pao)_2_(phen)]Cl·2H_2_O (**1**·2H_2_O) and [Co^III^(pao)_2_(bpy)]Cl·1.5MeOH (**2**·1.5MeOH). The structures of the complexes were determined by single-crystal X-ray crystallography. The Co^III^ ions are six-coordinate, surrounded by three bidentate chelating ligands, that is, two pao^−^ and one phen or bpy. The deprotonated oxygen atom of the pao^−^ ligand remains uncoordinated and participates in hydrogen bonding with the solvate molecules. IR data of the complexes are discussed in terms of the nature of bonding and the known structures.

## 1. Introduction

Oximes and their metal complexes are of current interest because of their rich physicochemical properties, reactivity patterns, and potential applications in many important chemical processes in the fields of medicine [1, 2], bioorganic chemistry [3], catalysis [4], and electrochemical and electrooptical sensors [5]. 

In the treatment of organophosphate insecticide toxicity in man and animals, the use of acetylcholinesterase reactivators in conjunction with atropine has been found to be the most effective treatment [6]. Among various acetylcholinesterase reactivators, 2-pyridinealdoxime (paoH) is routinely used in human and veterinary practices. It is furthermore known that metal complexes of active drugs as ligands can have important pharmaceutical activities because of several factors. In fact, the field of medicinal inorganic chemistry emerged a long time ago [7], and it is based on certain principles that can be summarized as follows. Complexation with the metal protects the drug against enzymatic degradations because of the inertness of certain metal-ligand linkages. The metal complex can have better hydrophobicity/hydrophilicity properties than the free ligand and, through this, it can improve the transport processes in the tissues. In addition, the metal complex can release the active drug(s) in a specific organ, and its activity can be reinforced by the combination of effects from the ligands and from the metal residue. The application of these principles has already resulted in the design of successful metal-based drugs [8, 9]. 

Since 1905, when Tschugaeff introduced dimethylglyoxime as a reagent for the analysis of nickel, oxime ligands have played an important role in the continuing progress of coordination chemistry [10]. Furthermore, the ability of the oximate(-1) group (>C = N–O^−^) to stabilize oxidized forms of metal ions, for example, Ni^III^ or Ni^IV^, has a vital importance in their role in the areas of technological applications [11]. In contrast to the great number of studies dealing with metal complexes of simple oximes and salicylaldoximes [12], relatively little is known about complexes of 2-pyridyloximes [13–15] although this class of compounds could offer unique features in terms of structural and physical properties. 

**Figure 1:**
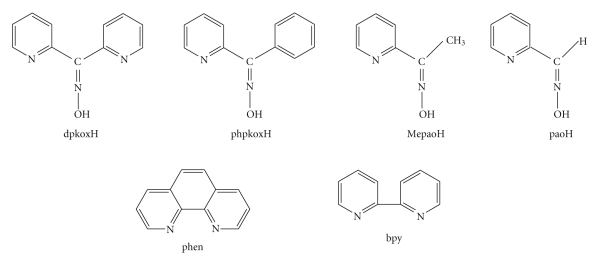
Structural formulae and abbreviations of the ligands discussed in the text.

Few years ago our group explored the influence of these ligands on the Co cluster chemistry by reacting cobalt carboxylate salts with neutral 2-pyridyloximes; the main objectives were the access to new structural types of clusters with interesting magnetic and spectroscopic properties and the study of Co-mediated reactions of the oxime group. Reaction schemes involving di-2-pyridyl ketone oxime, dpkoxH (Figure 1), and various Co carboxylate sources led [16] to the first mixed-valence Co(II,III), inverse 12-metallacrown-4 complexes, namely, [Co_2_^II^Co_2_^III^(OR)_2_(O_2_CR′)_2_(dpkox)_4_S_2_]X_2_ (R = H, CH_3_; *R*′ = CH_3_, C_6_H_5_, (CH_3_)_3_; S = solvent; X = ClO_4_, PF_6_). The use of phenyl 2-pyridyl ketone oxime, phpkoxH (Figure 1), methyl 2-pyridyl ketone oxime, MepaoH (Figure 1), and 2-pyridinealdoxime, paoH (Figure 1) in Co carboxylate chemistry yielded [17] the trinuclear, mixed-valence, carboxylate-free Co(II,III) complexes [Co_3_(phpkox)_6_](PF_6_)_2_, [Co_3_(Mepao)_6_](ClO_4_)_2_, and [Co_3_(pao)_6_](ClO_4_)_2_. The core of these complexes has an open-topology comprising one Co^II^ center and two Co^III^ ions. Recently [18] two of us reported complexes [Co_3_^III^O(O_2_CPh)_3_(pao)_3_]_2_[Co_3_^II^(O_2_CPh)_8_], [Co_3_^III^O(O_2_CPh)_3_(pao)_3_](O_2_CPh), [Co_3_^III^O(O_2_CPh)_3_(pao)_3_]_2_(O_2_CPh)(NO_3_), and [Co_2_^III^Co^II^(OH)Cl_3_(pao)_4_]. The above prior results encouraged us to proceed to the amalgamation of 2-pyridyl oximes with N,N′-donor ligands, that is, 1,10-phenanthroline (phen) and 2,2′-bipyridine (bpy) (Figure 1), in cobalt chemistry. The primary aim of this project was the synthesis and structural characterization of a series of cobalt building blocks that could act as “metalloligands” for the construction of one-dimensional heterometallic assemblies with various transition metal ions (e.g., Mn^III^, Cr^III^, Fe^II^) or lanthanides; the latter chains would probably provide interesting magnetic, optical, and spectroscopic properties. A secondary goal was the study of the biological properties of the resulting mixed-ligand complexes. Herein, we concentrate on the synthetic investigation of the general CoCl_2_/paoH/phen or bpy reaction system and describe the preparation and characterization of the cationic mononuclear complexes [Co(pao)_2_(phen)]Cl and [Co(pao)_2_(bpy)]Cl.

## 2. Experiments

### 2.1. Starting Materials and Physical Measurements

All manipulations were performed under aerobic conditions using reagents and solvents as received. Cobalt(II) chloride, 2-pyridinealdoxime (paoH), 1,10-phenanthroline hydrate (phen·H_2_O), and 2,2′-bipyridine (bpy) were purchased from Aldrich Co. Elemental analyses (C, H, N) were performed by the University of Ioannina (Greece) Microanalytical Laboratory using an EA 1108 Carlo Erba analyzer. Ir spectra (4000–450 cm^−1^) were recorded on a Perkin-Elmer 16 PC FT-IR spectrometer with samples prepared as KBr pellets. Solid-state (diffuse reflectance, 28.5–12.5 kK) electronic spectra were recorder on a Varian Cary 100 instrument. Magnetic susceptibility measurements were carried out at 25°C by the Faraday method using a Cahn-Ventron RM-2 balance standardized with [HgCo(NCS)_4_]*_n_*.

### 2.2. Compound Preparation

#### 2.2.1. Preparation of [Co(pao)_2_(phen)]Cl·2H_2_O (**1**·2H_2_O)

To a pale yellow, stirred solution of paoH (0.12 g, 1.0 mmol) in MeOH (20 cm^3^) was added a colourless solution of phen·H_2_O (0.10 g, 0.5 mmol) in the same solvent (5 cm^3^). To the resulting, almost colourless solution, a pink solution of CoCl_2_ (0.07 g, 0.5 mmol) in MeOH (10 cm^3^) was added. The deep orange solution obtained was stirred at ambient temperature for 30 min and allowed to very slowly evaporate at 6–8°C for one week. Well-formed, X-ray quality crystals of the product slowly appeared. The orange prismatic crystals were collected by filtration, washed with cold MeOH (2 × 3 cm^3^) and Et_2_O (2 × 4 cm^3^), and dried in air. Yields as high as 70% were obtained (found: C, 51.9; H, 3.8; N, 15.6. C_24_H_22_CoN_6_O_4_Cl calcd.: C, 52.1; H, 4.0; N, 15.2%).

#### 2.2.2. Preparation of [Co(pao)_2_(bpy)]Cl·1.5MeOH (**2**·1.5MeOH)

To a pink, stirred solution of CoCl_2_ (0.07 g, 0.5 mmol) in MeOH (15 cm^3^) was added solid paoH (0.12 g, 1.0 mmol). To the resulting deep orange solution a colourless solution of bpy (0.08 g, 0.5 mmol) in the same solvent (5 cm^3^) was added. The solution was stirred at ambient temperature for 25 min, filtered, and the filtrate was layered with Et_2_O/n-hexane (40 cm^3^, 1 : 1 v/v). Slow mixing gave well-formed, X-ray quality crystals of the product. The reddish orange prismatic crystals were collected by filtration, washed with cold MeOH (2 × 3 cm^3^) and Et_2_O (2 × 3 cm^3^), and dried in air. Yields as high as 75% were obtained. The crystals were found to lose solvent readily; the dried sample analysed for [Co(pao)_2_(bpy)]Cl, that is, **2** (found: C, 53.3; H, 3.4; N, 17.2. C_22_H_18_CoN_6_O_2_Cl calcd.: C, 53.6; H, 3.7; N, 17.1%).

### 2.3. X-Ray Crystallographic Studies [1]

Suitable crystals of **1**·2H_2_O were sealed in capillary filled with drops of the mother liquor, while crystals of **2**·1.5MeOH were mounted in air and covered with epoxy glou. Diffraction measurements of **1**·2H_2_O and **2**·1.5MeOH were made on a Crystal Logic dual goniometer diffractometer using graphite-monochromated Mo radiation. Crystal data and full details of the data collection and data processing are listed in Table 1. Unit cell dimensions were determined and refined by using three angular settings of 25 automatically centred reflections in the range 11° < 2*θ* < 23° for both complexes. Three standard reflections, monitored every 97 reflections, showed less than 3% intensity variation and no decay. Lorentz-polarisation corrections were applied for **1**·2H_2_O and **2**·1.5MeOH using Crystal Logic software.

The structures were solved by direct methods using SHELXS-97 [19] and refined by full matrix least-squares on *F* ^2^ with SHELXL-97 [20]. For both structures, all non-H atoms were refined using anisotropic thermal parameters. Some H-atoms were located by difference maps and refined isotropically. No H-atoms for the solvate molecules of **1**·2H_2_O were included in the refinement. 

Reference [1] CCDC codes are 771333 and 771334 for complexes **1**·2H_2_O and **2**·1.5MeOH, respectively.

## 3. Experiments

### 3.1. Synthetic Comments

The reaction system that we investigated was the CoCl_2_/paoH/phen or bpy one. Treatment of a solution of CoCl_2_ with two equivalents of paoH and one equivalent of 1,10-phenanthroline (phen) in MeOH, *under aerobic conditions*, followed by slow evaporation of the resulting solution allowed orange crystals of the product to be obtained in a form suitable for crystallography. The product was identified as the cationic mononuclear complex [Co(pao)_2_(phen)]Cl·2H_2_O (**1**·2H_2_O). Following a similar reaction scheme and replacing only the corresponding bidentate ligand with 2,2′-bipyridine (bpy), we were able to obtain red crystals suitable for crystallography. The new product was identified as [Co(pao)_2_(bpy)]Cl·1.5MeOH (**2**·1.5MeOH).

The most noticeable feature of this reaction scheme is the deprotonation of the oximato group without the presence of a strong base in the reaction system. Reduction products of the atmospheric oxygen, which is responsible for Co^II^ → Co^III^ oxidation, are possible agents for the former deprotonation. The formation of **1** and **2** can be summarized by the stoichiometric equations (1) and (2), respectively,

(1) $$\begin{array}{cc} & 2{\text{CoCl}}_{2}\;+4\text{paoH}+2\text{phen}\cdot {\text{H}}_{2}\text{O}+1/2\;{\text{O}}_{2}\\ & \;\;\overset{\;\text{MeOH}}{\to }2\left[\text{Co}{\left(\text{pao}\right)}_{2}\left(\text{phen}\right)\right]\text{Cl}+2\text{HCl}+3{\text{H}}_{2}\text{O} \end{array}$$

(2) $$\begin{array}{cc} & 2{\text{CoCl}}_{2}+4\text{paoH}+2\text{bpy}+1/2\;{\text{O}}_{2}\\ & \;\;\overset{\;\text{MeOH}}{\to }2\left[\text{Co}{\left(\text{pao}\right)}_{2}\left(\text{bpy}\right)\right]\text{Cl}+2\text{HCl}+{\text{H}}_{2}\text{O} \end{array}$$

The following experimental points should be mentioned at this point. (a) The reactions between CoCl_2_, paoH and N,N′-donor ligands in MeOH are [OH^−^]-independent. Addition of one equivalent of LiOH · H_2_O in the above described reaction mixtures leads to complexes 1 · 2H_2_O and 2 · 1.5MeOH as well as to unidentified noncrystalline, hydroxo compounds. (b) A number of attempts were made for the isolation of new products by increasing or decreasing the paoH/Co reaction ratio, keeping constant (1 : 1) the phen or bpy : Co ratio. Increasing the former ratio, that is, to 3 : 1 or 4 : 1, complexes **1** and **2** remained the main products; however, these were contaminated with variable amounts of the known product [Co^III^(pao)_3_] [21] (analytical evidence, unit cell determination of the isolated dark orange crystals). Reducing the above reaction ratio (1 : 1 and/or 0.5/1), paoH-“free” products were isolated containing only bpy or phen and chloride ions. We have not yet found evidence for the existence of the mixed-ligand species [Co(pao)(phen)_2_]Cl_2_ and [Co(pao)(bpy)_2_]Cl_2_. (c) Both complexes are soluble in water, dimethylformamide, dimethylsulfoxide, and acetonitrile, less soluble in nitromethane and ethanol, and insoluble in benzene, chloroform and dichloromethane.

### 3.2. Description of Structures

**Figure 2:**
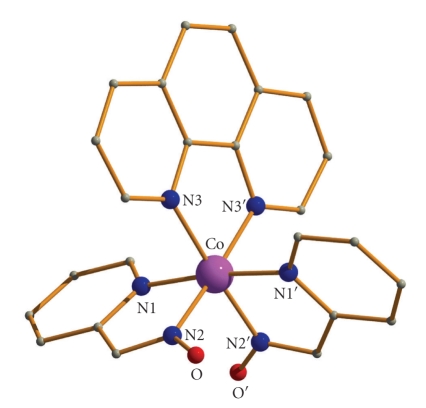
Partially labeled plot of the molecular structure of the cation that is present in **1**·2H_2_O. Primes are used for symmetry related atoms. H atoms have been omitted for clarity.

Labeled ORTEP plots of complexes **1**·2H_2_O and **2**·1.5MeOH are shown in Figures 2 and 4, respectively. Selected bond distances and angles for complexes **1**·2H_2_O and **2**·1.5MeOH are listed in Table 2. 

Disregarding the different nature of the N,N′-donor ligands, compounds **1**·2H_2_O and **2**·1.5MeOH display strikingly similar molecular structures. Thus, only the structure of the former will be described in detail.

Complex **1**·2H_2_O crystallizes in the tetragonal space group *I*4_1_/*acd*. Its structure consists of the mononuclear [Co(pao)_2_(phen)]^+^ cation, one chloride ion, and two solvate water molecules; the latter three will not be further discussed. The metal ion lies on a crystallographic 2-fold axis. The Co^III^ center is in a six-coordinate ligand environment comprising the two nitrogens from two chelating, anionic pao^−^ligands, [N(1)/N(2) and their symmetry-related partners] and two aromatic nitrogens from the chelating phen molecule [N(3) and N(3′)]. The metal coordination geometry is well described as distorted octahedral, its chromophore being Co^III^N_6_. Two *trans *positions of the octahedron are occupied by the pyridyl nitrogen atoms of the two pao^−^ ligands [N(1)–Co–N(1′) = 173.8(3)°]; thus, the two oximate nitrogen atoms [N(2), N(2′)] are in *cis* position. Angular distortions from perfect octahedral geometry are primarily a consequence of the chelating rings and their restricted bite angles. The Co–N bond lengths agree well with values expected for low-spin Co^III^ in octahedral environments [21].

**Figure 3:**
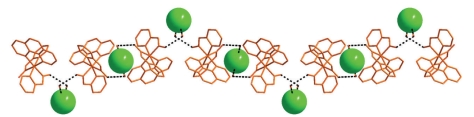
A part of the 1D structure of **1**·2H_2_O due to H-bonding interactions (dashed lines) along the *a* axis. Chlorides (green) are emphasized using space filling models.

**Figure 4:**
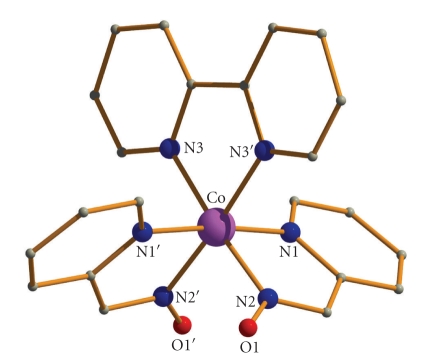
Partially labeled plot of the molecular structure of the cation that is present in **2**·1.5MeOH. H atoms have been omitted for clarity. Primes are used for symmetry related atoms.

In the crystal lattice of **1**·2H_2_O, the molecules interact through hydrogen bonds forming 1D zig-zag chains (Figure 3). These include the water lattice molecules, the oximate oxygen atom, and the Cl^−^ counteranion; their dimensions are presented in Table 3. 

**Figure 5:**
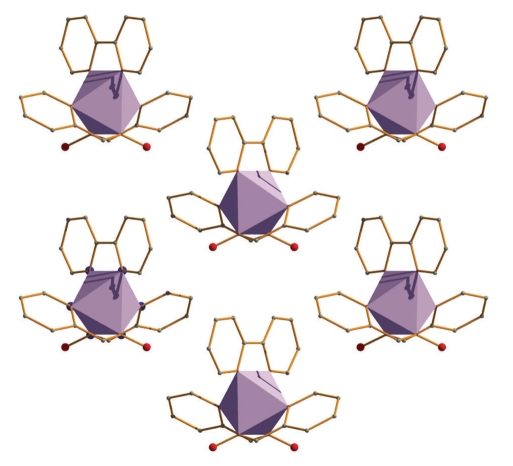
Packing diagram of complex **2**·1.5MeOH along the *c* axis. The metal coordination spheres are polyhedron designed (purple). The chloride counteranions and the methanol solvate molecules have been omitted.

Complex **2**·1.5MeOH crystallizes in the orthorhombic space group *Ic*2*m*; the metal ion lies on a crystallographic twofold axis. One half of the molecule comprises the asymmetric unit of the structure. The three ligands are N,N′-bidentate chelating. Again the pyridyl nitrogen atoms of the two pao^−^ ligands are in *trans* positions. A packing diagram of the complex is shown in Figure 5.

Since the space group of **1**·2H_2_O is centrosymmetric and the space group of **2**·1.5MeOH involves a mirror plane, both complexes are racemic mixtures of their Δ and Λ enantiomorphs. 

Complexes **1** and **2** join a small family of structurally characterized homo- [17, 18, 21] and heterometallic [22, 23] Co complexes featuring pao^−^ as ligand. Many years ago Blackmore and Magee [24, 25] and Grant and Magee [26] studied the reactions between 2-pyridinealdoxime and various Co^II^ sources under a variety of reaction conditions. Structural assignments of the solid products [24, 26] were based on spectroscopic data; no X-ray structures were reported. The authors did not prepare mixed-ligand complexes.

### 3.3. Physical and Spectroscopic Characterization

Complexes **1** and **2** are diamagnetic, in accordance with their low-spin 3d^6^ character.

The solid-state (diffuse reflectance) UV-Vis spectra of the two complexes are almost identical and typical for low-spin Co^III^N_6_ chromophores [27]. The low-spin octahedral ground term is ^1^ *A*_1*g*_, and there are two relatively low lying spin allowed transitions, with lower lying spin triplet partners, all derived from (*t*_2*g*_)^5^(*e*_*g*_). Under this scheme, the bands in the spectra of **1** and **2** at ~29.0, 21.5, 17.0, and 13.5 kK are assigned [27] to the ^1^ *A*_1*g*_→^1^ *T*_2*g*_, ^1^ *T*_1*g*_, ^3^ *T*_2*g*_, and ^3^ *T*_1*g*_ transitions, respectively, although a superposition of the highest energy d-d transition and a charge transfer band should not be ruled out.

Two bands, one of medium intensity at ~1015 cm^−1^ assigned to **ν**(N–O) and one strong at 1599 cm^−1^ assigned to *ν*(C=N)__oximate_ are common in the IR spectra of the two complexes [21]; the higher-wavenumber band most probably overlaps with an aromatic stretch. The in-plane deformation of the 2-pyridyl ring of the free paoH at 627 cm^−1^ shifts upward on coordination in the spectra of **1** (643 cm^−1^) and **2** (645 cm^−1^) [28].

## 4. Conclusions

The present work extends the body of results that emphasizes the ability of the monoanionic ligand pao^−^ to form interesting structural types in 3d metal chemistry. The use of both paoH and phen or bpy in reactions with Co^II^ sources has led to products **1** and **2**, the first mixed-ligand Co^III^ noncarboxylate complexes involving paoH/pao^−^. Of interest is the nonparticipation of the deprotonated oximate oxygen atom in coordination; this is due to the involvement of the negatively charged oxygen in hydrogen bonding.

Complexes of trivalent 3d metals (e.g., Cr^III^, Mn^III^, Fe^III^) other than Co^III^ with the pao^−^/phen or bpy ligand combinations are not known to date, and it is currently not evident whether the structures of such compounds are dependent on the particular nature of the metal ion. We are studying this matter. Synthetic efforts are also in progress to use **1** and **2** as “metalloligands” for the preparation of heterometallic Co^III^/M^III^ complexes (M = Fe, Mn, lanthanides).

## Figures and Tables

**Table 1:** Crystallographic data for complexes **1**·2H_2_O and **2**·1.5MeOH.

Parameter	**1**·2H_2_O	**2**·1.5MeOH
Color (habit)	Red/orange prisms	Red prisms
Crystal size (mm)	0.33 × 0.08 × 0.05	0.50 × 0.35 × 0.30
Chemical formula	C_24_H_22_CoN_6_O_4_Cl	C_23.5_H_19_CoN_6_O_3.5_Cl
*M*	552.86	535.83
Crystal system	Tetragonal	Orthorhombic
Space group	*I*4_1_/*acd *	*Ic*2*m *
Unit cell dimensions		
*a* (Å)	17.098(5)	13.134(6)
*b* (Å)	17.098(5)	8.900(4)
*c* (Å)	31.903(9)	21.293(8)
*V* (Å^3^)	9327(5)	2489(2)
*Z*	16	4
*D*_calc_ (g cm^−3^)	1.575	1.430
*μ* (mm^−1^)	0.896	0.836
Radiation (Å)	Mo*K*_*α*_ (0.71073)	Mo*K*_*α*_ (0.71073)
Temperature (K)	298	298
Scan mode/speed (°min^−1^)	*θ*-2*θ*/2.6	*θ*-2*θ*/3.5
Scan range (°)	2.1 + *α*_1_ *α*_2_ separation	2.3 + *α*_1_ *α*_2_ separation
*θ* range (°)	2.1–23.0	1.8–25.0
Reflections collected	3157	3561
Unique reflections	1627 (*R*_int_ = 0.0276)	2253 (*R*_int_ = 0.0240)
Reflections used [*I* > 2*σ*(*I*)]	1237	1882
Parameters refined	164	204
[Δ/*σ*]_max _	0.000	0.014
[Δ*ρ*]_max _/[Δ*ρ*]_min_ (e Å^−3^)	0.712/−0.444	0.695/−0.244
GoF (on *F* ^2^)	1.101	1.079
*R*_1_^(a)^ [*I *> 2*σ*(*I*)]	0.0543	0.0424
*w* *R*_2_^(b)^ [*I *> 2*σ*(*I*)]	0.1453	0.1097

^(a)^ *R*_1_= Σ(|*‌* *F*_*o*_ | *‌*−|*‌* *F*_c_ | *‌*)/Σ(|*‌* *F*_*o*_ | *‌*); ^(b)^ *w* *R*_2_  = {Σ[*w*(*F*_*o*_^2^−*F*_c_^2^)^2^]/Σ[*w*(*F*_*o*_^2^)2]}^1/2^, *w* = 1/[*σ* ^2^(*F*_*o*_^2^) + (*a* *P*)^2^ + *b* *P*] where *P * = (max(*F*_*o*_^2^, 0) + 2*F*_c_^2^)/3.

**Table 2:** Selected bond distances (Å) and angles (°) for complexes **1**·2H_2_O and **2**·1.5MeOH,^a^ with the estimated standard deviations in parentheses.

	**1**·2H_2_O	**2**·1.5MeOH
*Bond distances*		
Co–N(1)	1.928(4)	1.937(3)
Co–N(2)	1.904(4)	1.909(4)
Co–N(3)	1.986(4)	1.977(4)
Co–N(1′)	1.928(4)	1.937(3)
Co–N(2′)	1.904(4)	1.909(4)
Co–N(3′)	1.986(4)	1.977(4)
N(2)–O/O(1)	1.294(6)	1.273(5)
N(3)–C(7)	1.322(7)	1.343(6)
N(1)–C(1)	1.343(7)	1.340(6)
N(2)–C(6)	1.298(7)	1.309(7)

*Bond angles*		
N(3)–Co–N(3′)	82.7(2)	82.2(2)
N(1)–Co–N(3)	88.6(2)	93.6(2)
N(1)–Co–N(2)	83.7(2)	83.7(2)
N(2)–Co–N(2′)	89.9(3)	89.8(2)
N(1)–Co–N(3′)	96.1(2)	90.7(2)
Co–N(2)–O/O(1)	122.7(4)	123.3(3)

^a^Unprimed and primed atoms are related by symmetry.

**Table 3:** Hydrogen bonding interactions in **1**·2H_2_O.

Interaction D–H⋯A	D⋯A (Å)	H⋯A (Å)	D–H⋯A (°)	Symmetry operation of A
OW-HWA⋯O	2.854(7)	1.740(4)	161.7(3)	*x*, *y*, *z *
OW-HWB⋯Cl	3.124(6)	1.978(2)	163.5(3)	*x*, *y*, *z *
